# Special Issue “Thyroid Dysfunctions: Molecular Mechanisms Underlying Clinical Consequences”

**DOI:** 10.3390/ijms262411967

**Published:** 2025-12-12

**Authors:** Jacopo Manso

**Affiliations:** Endocrinology Unit, University Hospital S. Maria della Misericordia of Udine, Oncology Area Deparment, Azienda Sanitaria Universitaria Friuli Centrale (ASUFC), 33100 Udine, Italy; jacopo.manso@asufc.sanita.fvg.it

The thyroid gland, and the hypothalamic–pituitary–thyroid (HPT) axis that governs it, represent a central nexus of metabolic and developmental control. Thyroid hormones (THs) are indispensable for neurodevelopment, systemic metabolism, and the function of virtually every organ system [1]. Consequently, thyroid dysfunction—spanning hypothyroidism and hyperthyroidism—remains one of the most prevalent endocrine disorders globally, affecting millions of patients.

While clinical management of these conditions, particularly hypothyroidism, has been established for decades, a significant gap often persists between biochemical normalization and the complete resolution of clinical symptoms. This gap highlights the profound complexity of TH action, which is not limited to circulating hormone levels but rather is dictated by intricate, tissue-specific molecular mechanisms, including transport, local activation, and receptor-mediated signaling. Understanding the precise molecular events that underlie the clinical consequences of thyroid dysfunction is paramount to advancing patient care and developing novel therapeutics.

It is with great pleasure that I introduce this Special Issue, “Thyroid Dysfunctions: Molecular Mechanisms Underlying Clinical Consequences,” a collection of five outstanding articles that delve into this complexity. These contributions span from fundamental neurobiology and genetic engineering to systemic pathophysiology and novel cancer therapeutics. I extend my sincere gratitude to all the authors for their high-quality submissions and to the many expert reviewers whose rigorous feedback ensured the scientific merit of this collection.

The articles herein can be broadly viewed across three essential themes: the fundamental mechanisms of TH action, systemic and pathophysiological regulation, and thyroid oncology.

## 1. Fundamental Mechanisms of Thyroid Hormone Action

Two articles in this collection provide insights into *how* and *where* thyroid hormones act, challenging long-held assumptions.

Graceffo et al. (**Contribution 1**) address the critical, yet poorly understood, regulation of thyroid hormone receptor alpha (THRA) in the human brain. Using advanced bulk and single-cell RNA-sequencing analyses, they demonstrate a strong and unexpected predominance of THRA2—a splicing isoform that lacks the ligand-binding domain and is thought to act as an inhibitor—in both the developing and adult human brain. This finding suggests that T3-mediated transcription in the central nervous system is tightly buffered, providing a crucial molecular context for understanding neurodevelopment and the incomplete resolution of neurological symptoms in some hypothyroid patients.

Complementing this, Citterio et al. (**Contribution 2**) present an elegant and innovative approach to dissecting T3 versus T4 action in vivo. They generated a novel knock-in mouse model (ChEL-KI) whose thyroid gland is genetically engineered to selectively secrete T3, rendering it T4-deficient. Despite profound T4 deficiency and high TSH, these mice maintain normal circulating T3 levels. The resulting phenotype is revelatory: hepatic markers of TH action (T3-dependent) are normalized, whereas behavioral and motor deficits (reliant on local T4-to-T3 conversion in the CNS) persist. This work provides evidence for the tissue-specific roles of T4 and T3 and offers a powerful preclinical model to explore the nuances of T4 monotherapy versus combination T4/T3 replacement.

## 2. Systemic and Pathophysiological Regulation

Moving from fundamental action to complex systemic interactions, two articles explore how thyroid function is integrated with, and profoundly affects, other major physiological systems.

Parra-Montes de Oca et al. (**Contribution 3**) investigate the long-term interplay between stress, exercise, and the HPT axis. In a comprehensive rat model, they demonstrate that chronic variable stress during adolescence permanently alters the HPT axis’s response to exercise in adulthood, and notably, this “reprogramming” is sex-dependent. Stress blunted the exercise-induced activation of the HPT axis and key peripheral metabolic genes (e.g., *Dio2*, *Pgc1a*). This study provides compelling evidence of the allostatic load that early life events can impose on endocrine circuits, with direct clinical implications for understanding metabolic and psychiatric comorbidities.

Da Silva et al. (**Contribution 4**) address a frequent clinical consequence of hypothyroidism: female infertility. Their work provides a novel molecular link, demonstrating that hypothyroidism in rats disrupts the estrous cycle by directly altering the uterine kisspeptin system (Kiss1/Kiss1r). This disruption was associated with impaired estrogen and progesterone signaling and reduced expression of uterine receptivity mediators, such as LIF and BMP2. This study identifies a specific pathway connecting thyroid status to reproductive health, offering new targets for investigation in the management of infertility.

## 3. Thyroid Pathology and Novel Therapeutics

Finally, this collection addresses thyroid dysfunction at the level of thyroid pathology itself. My own group, Manso et al. (**Contribution 5**), explores novel therapeutic avenues for papillary thyroid cancer (PTC), the most common endocrine malignancy. We investigated the anti-cancer properties of licorice extract (LE) and its major component, glycyrrhetinic acid (GA), on PTC cell lines. Our findings show that both LE and GA significantly inhibit cell proliferation and migration. Mechanistically, we found that LE primarily induces oxidative stress, whereas GA induces apoptosis, and these effects are not mediated solely via the mineralocorticoid receptor. This work identifies natural compounds as a new source for the development of new therapeutic strategies for PTC.

Together, these five articles underscore the dynamism and breadth of modern thyroid research. They move the field beyond simple measurements of circulating hormones and into the complex molecular environment of specific tissues—from the splicing of a single receptor gene in the brain (**Contribution 1**) and the genetic engineering of T3-specific secretion (**Contribution 2**) to the intricate feedback loops involving stress (**Contribution 3**) and reproduction (**Contribution 4**), and ultimately to the search for new treatments for thyroid cancer (**Contribution 5**).

The future of endocrinology lies in this multi-level integration. I trust that this Special Collection will serve as a valuable resource and inspire further research to bridge the critical gap between these profound molecular mechanisms and their tangible clinical consequences for the patients we treat.
